# Biosurfactants as Stabilizers of Silver Nanoparticles: A Sustainable Approach for Antimicrobial Applications

**DOI:** 10.3390/microorganisms14061379

**Published:** 2026-06-22

**Authors:** Renata R. Silva, Hugo M. Meira, Marcos Antonio B. Lima, Jaciana dos S. Aguiar, Leonie A. Sarubbo, Juliana M. Luna

**Affiliations:** 1 Northeast Biotechnology Network (RENORBIO), Federal Rural University of Pernambuco, Dom Manuel de Medeiros Street, Dois Irmãos, Recife 52171-900, Brazil; renatabiology2015@gmail.com; 2 Advanced Institute of Technology and Innovation (IATI), Rua Potira, n. 31—Prado, Recife 50070-280, Brazil; hugomorais09@gmail.com (H.M.M.); leonie.sarubbo@unicap.br (L.A.S.); 3 Postgraduate Program in Chemical Engineering, Federal University of Pernambuco, Professor Moraes Rego Avenue, n. 1235—Cidade Universitária, Recife 50670-901, Brazil; 4 Postgraduate Program in Environmental Process Development, Catholic University of Pernambuco (UNICAP), Rua do Príncipe, n. 526—Boa Vista, Recife 50050-900, Brazil; marcos.barbosalima@ufrpe.br; 5 Center of Biological Sciences, Department of Antibiotics, Federal University of Pernambuco, Av. Prof. Artur de Sá, Cidade Universitária, Recife 54740-520, Brazil; jaciana.aguiar@ufpe.br; 6 School of Technology and Communication, Catholic University of Pernambuco (UNICAP), Rua do Príncipe, n. 526—Boa Vista, Recife 50050-900, Brazil; 7 School of Health and Life Sciences, Catholic University of Pernambuco (UNICAP), Rua do Príncipe, n. 526—Boa Vista, Recife 50050-900, Brazil

**Keywords:** microbial surfactant, antibiotics, resistance, microorganisms, BS-AgNPs

## Abstract

Microbial resistance to conventional antimicrobials is a growing public health challenge, driving the search for effective and sustainable alternatives. Among emerging strategies, the combination of silver nanoparticles (AgNPs), recognized for their potent antimicrobial action, with biosurfactants, natural, biodegradable compounds capable of interacting with microbial cell membranes and promoting their stabilization stands out. In this context, the aim of this study was to produce a biosurfactant by *Candida glabrata* UCP 1002 from agroindustrial residues, reducing costs and environmental impacts. The compound exhibited a surface tension of 29 mN/m, a critical micellar concentration of 0.3%, and a yield of 9 g/L; furthermore, it demonstrated stability across wide ranges of temperature, pH, and salinity. The AgNPs were synthesized using the biosurfactant as a stabilizing agent and ascorbic acid as a reducing agent, resulting in stable particles. In antimicrobial assays, the formulation inhibited Gram-positive microorganisms, Gram-negative microorganisms, and fungi. The best results were obtained against *Pseudomonas aeruginosa* (26.63%) and *Candida albicans* (28.11%), followed by *Staphylococcus aureus* (17.58%), *Enterobacter* sp. (14.42%), and *Escherichia coli* (13.68%). Although less effective than commercial antibiotics such as streptomycin and moxifloxacin, it showed potential as a complementary alternative in combating multidrug-resistant pathogens. Cytotoxicity assays revealed low toxicity toward normal cells (28.42% inhibition in Vero CCL-81) and minimal activity against tumor cells. The results demonstrate that the BS-AgNPs association combines relevant antimicrobial activity with environmental safety and biocompatibility, establishing itself as a promising and sustainable approach for application in health, industry, and the environment, with potential for scale-up production from low-cost raw materials.

## 1. Introduction

The accelerated growth of bacterial and fungal resistance to antimicrobial agents, driven by genetic mutations or the incorporation of exogenous genes, constitutes a significant threat to public health. Over the past two decades, the emergence of resistant pathogens has become a serious threat to the healthcare sector. Infections caused by *Enterococcus faecium*, *Staphylococcus aureus*, *Klebsiella pneumoniae*, *Acinetobacter baumannii*, *Pseudomonas aeruginosa*, and *Enterobacter* spp., known as ESKAPE pathogens, represent a significant challenge, as they can exhibit multiple resistance mechanisms and transfer them through horizontal gene transfer [1]. Several environmental sources have been identified as reservoirs of antimicrobial-resistant bacteria, including underground cisterns used for the storage of human waste, surfaces and medical devices in clinical settings, as well as hospital and municipal sewage systems [2].

Given this scenario, the discovery and development of new antibacterial substances capable of inhibiting the emergence and spread of microbial resistance has become urgent [3]. One of the strategies suggested to contain the advance of antibiotic-resistant infections is the combination of antimicrobial agents with other compounds, such as surfactants [4]. Various types of surfactants can interact with the cell membranes of microorganisms through electrostatic and hydrophobic interactions, and are widely used as antimicrobial agents in different sectors, such as the healthcare and food industries [3].

Chemical surfactants, although widely used in industrial sectors, present serious environmental and toxicological challenges, such as bioaccumulation, low biodegradability, algal bloom formation, and microbial resistance. In response to these limitations, biosurfactants have been promoted as green, natural, and biodegradable alternatives, with promising applications in the pharmaceutical, food, and cosmetic industries. Furthermore, innovative strategies are being developed to improve the production and efficiency of these compounds, aiming to reduce environmental impacts and advance sustainable technologies [5,6,7,8].

The specific antimicrobial mechanisms of biosurfactants have not yet been fully elucidated. However, it is believed that these compounds act primarily through interactions with bacterial cell membranes, although their action is not restricted to a single mechanism. Another hypothesis is that the alkyl chains of surfactants interact hydrophobically with the lipid bilayer, promoting disorganization of the membrane structure. This disruption favors the leakage of intracellular constituents, leading to loss of cellular integrity and, ultimately, to bacterial cell death [9,10,11,12].

Silver nanoparticles (AgNPs) have been widely used in various applications due to their inorganic nature, low toxicity, and remarkable antimicrobial properties. For their characteristics to be adjusted according to the desired purpose, it is essential to understand the requirements involved in the manufacturing processes, enabling the controlled modification of the synthetic elements used. AgNPs are widely incorporated into consumer products such as detergents, toothpastes, foods, textiles, and clothing, standing out for their effective antibacterial and antifungal activity against a wide variety of microorganisms [13]. Furthermore, the use of biosurfactants as reducing and stabilizing agents in the green synthesis of silver nanoparticles has emerged as an innovative approach to enhance the antimicrobial activity of these nanoparticles, exploring the synergistic potential between both compounds [14].

Nanotechnology, especially green nanotechnology, offers promising applications in the detection and treatment of diseases, while also enabling the development of more effective strategies to potentiate the action of drugs and antimicrobial agents. At the nanoscale, surface effects become predominant, increasing the surface energy of the nanoparticles. To reduce this energy, they tend to aggregate, which can compromise the stability of the system. Biosurfactants represent an effective and sustainable approach to addressing nanoparticle instability issues. Their unique properties, combined with biocompatibility and environmental benefits, make them promising alternatives for enhancing the stability and functionality of nanoparticles in industrial, medical, and environmental applications. As a result, biosurfactants emerge as important allies for the responsible and sustainable use of nanotechnology as this field continues to evolve [13,15].

Among the most relevant properties of biosurfactants is their capacity to interact with biological membranes. Through insertion into the lipid bilayer, these compounds promote increased membrane permeability and destabilization of electrochemical gradients, resulting in the uncontrolled leakage of intracellular metabolites and, consequently, cell lysis. Such characteristics confer on biosurfactants a broad potential for application in industrial and scientific contexts, with particular emphasis on the development of medical and pharmacological therapies [16].

In this context, the present work aimed to synthesize and characterize silver nanoparticles (AgNPs) using the biosurfactant produced by *Candida glabrata* UCP 1002 as stabilizing agent, exploring the synergistic potential between both compounds, as well as to evaluate the antimicrobial activity of the formulations obtained against clinical isolates, including pathogens of clinical relevance.

## 2. Materials and Methods

### 2.1. Biosurfactant Production

*Candida glabrata* UCP 1002, obtained from the culture collection of the Catholic University of Pernambuco (UCP), Pernambuco, Brazil, was cultivated in a medium containing distilled water supplemented with 2.5% residual frying oil, 2.5% corn steep liquor, and 2.5% molasses. The pH of the medium was adjusted to 5.5, followed by incubation with a cell suspension of 10^7^ cells/mL in a shaker at 200 rpm and 28 °C for 144 h, based on previously established conditions [17].

### 2.2. Determination of Surface Tension and Critical Micellar Concentration

To confirm biosurfactant production, the cell-free metabolic liquid was subjected to surface tension determination using a tensiometer with a Du Noüy ring (KSV Instruments Ltd., Sigma 700, Helsinki, Finland). The force required to pull the ring through the liquid/air interface was recorded [17].

### 2.3. Determination of Critical Micellar Concentration (CMC)

The critical micellar concentration (CMC) was calculated by measuring the surface tension of biosurfactant dilutions in distilled water until a constant surface tension was obtained. The CMC was derived from a graph of surface tension versus biosurfactant concentration and expressed in g/L [17].

### 2.4. Biosurfactant Extraction

The biosurfactant was isolated using a ratio of 1/4 of crude metabolic liquid to solvent (ethyl acetate). The procedure was performed twice, followed by centrifugation of the solvent at 4500 rpm for 15 min. The organic phase was placed in a separatory funnel, and the sample was washed with a saturated sodium chloride (NaCl) solution so that any aqueous phase that subsequently appeared could be discarded. Finally, the solvent was dried with sodium sulfate [17].

### 2.5. Evaluation of Biosurfactant Stability

The stability of the produced biosurfactant was evaluated under three environmental conditions: temperature, salinity, and pH. To this end, the effects of different temperatures (5 °C, 28 °C, 70 °C, and 100 °C), NaCl concentrations (2%, 4%, 6%, 8%, 10%, and 12%), and pH ranges (2, 4, 6, 8, 10, and 12) were analyzed separately. The biosurfactant was then subjected to surface tension analyses to assess its stability. All assays were performed in triplicate.

### 2.6. Fourier-Transform Infrared Spectroscopy (FT-IR)

The chemical composition and structure of the semi-purified biosurfactant were characterized by Fourier Transform Infrared Spectroscopy (FTIR) using a Spectrum 400 instrument (Perkin Elmer, Shelton, CT, USA) [17].

### 2.7. Synthesis of Biosurfactant Stabilized Silver Nanoparticles

Silver nanoparticles were synthesized by a laboratory developed method using biosurfactant as a stabilizing agent and ascorbic acid as a reducing agent. Initially, an aqueous solution of silver nitrate at 0.9 g/L was prepared, corresponding to a final concentration of 500 ppm of metallic silver. Subsequently, 2.8 g/L of biosurfactant was added to the AgNO_3_ solution under constant stirring on a magnetic stirrer at room temperature until complete solubilization. Thereafter, ascorbic acid at a proportion of 1.3 g/L was added slowly and continuously, with the mixture kept under constant stirring for one hour. During the process, nanoparticle formation was monitored visually by the color change in the solution (from colorless to shades of yellow to brown), characteristic of AgNP formation. After synthesis, the solution was kept at rest at room temperature for nanoparticle stabilization.

### 2.8. Properties and Characteristics of Silver Nanoparticles by SEM and EDS

The morphological characterization of biosurfactant-stabilized silver nanoparticles was performed by Scanning Electron Microscopy (SEM) using FEI QUANTA 200F equipment for image acquisition and TESCAN Mira 4 for analysis by Energy Dispersive X-ray Spectroscopy (EDS). Initially, the sample was fixed on an aluminum stub appropriate for SEM using double-sided self-adhesive conductive carbon tape. Subsequently, the samples were stored in a desiccator until the time of analysis.

Analyses were performed by directing a focused electron beam onto the sample surface in scanning mode under high vacuum conditions. For image acquisition, an acceleration voltage of 20 kV, spot size 4, and working distance ranging from 7.3 mm to 7.6 mm were used according to the image obtained. Everhart Thornley Detector (ETD) secondary electron detectors and Backscattered Electrons (BSE) detectors were employed, with a resolution of 1024 × 896 pixels.

Subsequently, elemental analysis of the samples was performed by EDS for identification of the surface chemical composition of the nanoparticles. Analyses were conducted in high vacuum mode using a beam acceleration voltage of 10 kV, probe current of 10 nA, and working distance of 15 mm. A TESCAN OEM detector (C2), serial number 005299, was used, with a spectral collection range between 0 and 20 keV and an acquisition resolution of 10 eV per channel. Elemental maps were obtained with a resolution of 512 × 512 pixels.

### 2.9. Properties and Characteristics by Zeta Potential and Particle Size by Dynamic Light Scattering (DLS)

The determination of the zeta potential and particle size by dynamic light scattering (DLS) of the biosurfactant-stabilized silver nanoparticles was performed using an Anton Paar Litesizer 500 Particle Analyzer, serial number 82649111, operated by Anton Paar Kalliope Professional software version 2.34.3.

For sample preparation, approximately 0.005 g of material was dispersed in 10 mL of PA n-hexane previously filtered through a syringe with a 0.22 µm PTFE membrane, aiming to minimize the presence of interfering particles. Subsequently, the samples were subjected to dispersion in an ultrasonic bath to promote homogenization and reduce possible agglomerates.

### 2.10. Antimicrobial Activity

Sterile filter paper disks (Whatman No. 1, 5 mm in diameter) were impregnated with the biosurfactant stabilized with silver nanoparticles (BS-AgNPs) and placed on the surface of Petri dishes previously inoculated with the target microorganisms: *Enterobacter* sp., *Staphylococcus aureus*, *Escherichia coli*, *P. aeruginosa* (bacterial strains isolated from clinical samples from a hospital), and *Candida albicans*. Mueller-Hinton agar was used for culturing the microorganisms. Plates with microorganisms were incubated at 72 °C for 24 h. After incubation, inhibition halos were measured and results expressed as percentage of inhibition, as described by Leyton et al. [18]. All assays were conducted in triplicate.

### 2.11. Cytotoxic Evaluation of the Biosurfactant

Cytotoxic activity was assessed using the MTT (3-(4,5-dimethylthiazol-2-yl)-2,5-diphenyltetrazolium bromide) method [19,20].

The cell lines HL-60 (human promyelocytic leukemia), HT-29 (human colon adenocarcinoma), MCF-7 (human breast adenocarcinoma), and Vero CCL-81 (African green monkey kidney epithelial cells) were maintained in DMEM or RPMI 1640 culture medium supplemented with 10% inactivated fetal bovine serum, 1% antibiotic solution (penicillin and streptomycin), and 1% L-glutamine. Cells were maintained in an incubator at 37 °C in a humidified atmosphere enriched with 5% CO_2_ and 95% humidity.

HT-29, MCF-7, Vero CCL-81 (10^5^ cells/mL), and HL-60 (3 × 10^5^ cells/mL) cells were added to 96-well plates and incubated for 24 h (except for the HL-60 line, which did not require 24 h of incubation), after which 10 µL of the solutions were added to the wells at a final concentration of 30 µg/mL (0.5% DMSO). Paclitaxel (10 µg/mL) was used as the standard drug. After 72 h of re-incubation, 25 µL of MTT (5 mg/mL) was added, and after 3 h of incubation, the culture medium containing MTT was aspirated and 100 µL of DMSO was added to each well. Absorbance was measured in a microplate reader at a wavelength of 560 nm.

Experiments were performed in quadruplicate and the percentage of inhibition was calculated using GraphPad Prism 7.0 demo. For substances that showed inhibition greater than 70%, the IC_50_ (concentration that inhibits 50% of growth relative to the negative control) and the respective confidence intervals (95% CI) were calculated from nonlinear regression using GraphPad Prism 7.0 demo.

### 2.12. Statistical Analyses

All tests were performed in triplicate and data are expressed as mean ± standard deviation. ANOVA analysis were used to determine significance.

## 3. Results and Discussion

### 3.1. Biosurfactant Production

**Figure 1:**
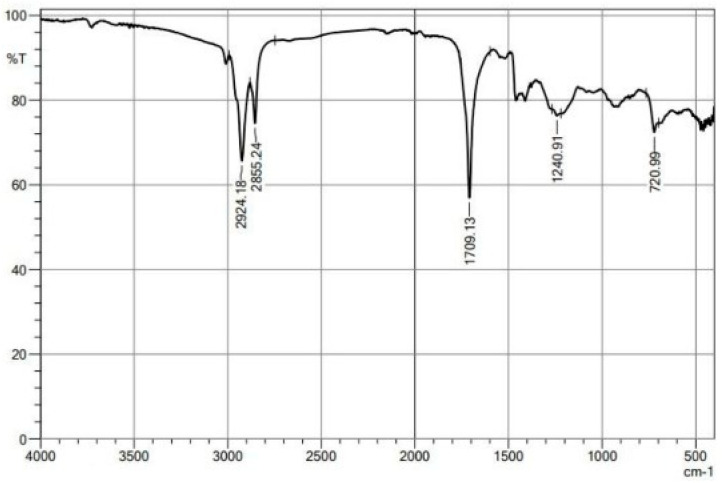
FTIR spectrum of the biosurfactant produced by *C. glabrata* UCP 1002, showing the main characteristic absorption bands.

After 144 h of cultivation at 200 rpm and 28 °C, the biosurfactant produced by *C. glabrata* UCP 1002 reduced the surface tension of water from 72 to 29 mN/m, the critical micellar concentration was reached at 0.3%, and, after the isolation process, a yield of 9.0 g/L was obtained. FTIR analysis revealed characteristic absorption bands consistent with lipid-based compounds. The strong absorption band at 1709.13 cm^−1^ is attributed to the stretching vibration of the carbonyl group (C=O), typically associated with carboxylic acids, esters, or ketones present in fatty acid derivatives. The bands at 2924.18 cm^−1^ and 2855.24 cm^−1^ correspond to asymmetric and symmetric stretching vibrations of C-H bonds in methylene (–CH_2_–) and methyl (–CH_3_) groups, respectively, indicative of long aliphatic hydrocarbon chains, which are widely reported in the characterization of lipidic biosurfactants (Figure 1) [17].

According to Farmadi et al. [21], several microorganisms possess the ability to synthesize and secrete biosurfactants with distinct physicochemical properties. Among the genera most frequently used in biotechnological processes, *Pseudomonas* spp., *Bacillus* spp., *Rhodococcus* spp., *Candida* spp., *Lactobacillus* spp., *Arthrobacter* spp., and *Acinetobacter* spp. stand out.

The substrates used in biosurfactant production can account for up to 30% of the total process cost. Therefore, the use of low-cost raw materials, such as residues from industrial and agricultural activities, emerges as a sustainable alternative for waste management. Agricultural, refinery, and food industry residues stand out as viable substitutes for more expensive inputs, contributing to the reduction in production costs and promoting greater economic and environmental sustainability. Among these, agricultural residues have been identified as the most promising, potentially reducing production costs by up to 10% [22,23,24,25,26].

Agri-food residues have a complex composition, consisting of polysaccharides, proteins, carbohydrates, polyphenolic compounds, and other constituents. They are considered low-cost natural resources, widely available, renewable, and environmentally sustainable. Various biotechnological solutions can be employed for their utilization, offering economically viable and easily applicable alternatives, with positive impacts on both environmental preservation and human health. Several agroindustrial residues and by-products can be used as substrates in biosurfactant production [27]. Produced by microorganisms from renewable resources, biosurfactants represent a viable and sustainable alternative to conventional surfactants of petrochemical origin [24,28,29].

Almeida and Sarubbo et al. [30] investigated the potential of the alkaliphilic bacterium *Pantoea* sp. to use a low-cost medium supplemented with vegetable fat (25%), corn steep liquor (5%), and pineapple peel broth (2%) for biosurfactant production. Biosurfactant production was conducted in a 5 L bioreactor at 30 °C for 72 h, under agitation of 350 rpm and aeration of 0.5 vvm. Synthesis began after 36 h, reaching a concentration of 1.25 g/L at 60 h. The biosurfactant exhibited low viscosity, reduced surface tension to 30 mN/m, and showed a CMC of 1.0 g/L.

Lira et al. [31] produced a biosurfactant from *C. guilliermondii*, cultivated in a medium with 5% molasses, 5% corn steep liquor, and 5% residual frying oil, and obtained a reduction in surface tension to 28.6 mN/m, a yield of 21 g/L, and a critical micellar concentration of 0.7 g/L.

Therefore, the results presented demonstrate the high potential of biosurfactants produced by different microorganisms, especially when cultivated in media containing agroindustrial residues. The significant reduction in surface tension and the good yields obtained reinforce the technical feasibility of these bioproducts. Furthermore, the use of low-cost substrates contributes to the economic and environmental sustainability of the process. In this context, biosurfactants emerge as promising alternatives to synthetic surfactants. Thus, their large-scale application tends to grow, driven by sustainable biotechnological solutions.

### 3.2. Evaluation of Biosurfactant Surface Tension Stability

Biosurfactants offer several advantages over synthetic surfactants, including greater tolerance to high temperatures, stability over a wide pH range, high resistance to salinity, superior biodegradability, low toxicity, and greater selectivity in their applications [32]. According to the literature, long-term stability is an essential requirement for the development and commercial viability of new biotechnological products, which must maintain their functionality even in the face of variations in pH, temperature, and salinity frequently encountered in industrial environments [33].

According to the results shown in Table 1, the evaluated biosurfactant maintained its ability to reduce surface tension even under significant variations in temperature, pH, and NaCl concentration, demonstrating physicochemical stability. Regarding temperature, a reduction from 34.1 mN/m (0 °C) to 32.2 mN/m (100 °C) was observed, indicating good thermal stability. As for pH, the best performance was recorded at pH 6, with a surface tension of 29.4 ± 0.07 mN/m, while the other values varied moderately between 31.4 and 34.9 mN/m, nonetheless confirming efficiency over a wide pH range (2–12). Regarding salinity, surface tension remained stable up to 8% NaCl (33.8 ± 0.1 mN/m), with a slight increase at 10% (35.2 ± 0.2 mN/m) and 12% (34.7 ± 0.5 mN/m), indicating good saline tolerance up to moderately high concentrations. These data reinforce the robustness and versatility of the biosurfactant, making it promising for industrial applications in environments with extreme variations in temperature, pH, and salinity.

Albuquerque et al. [34] evaluated the surface tension stability of a biosurfactant produced by *Bacillus subtilis* UCP 1533. Regarding temperature, surface tension remained low throughout the entire analyzed range (5–100 °C), with the lowest value recorded at 50 °C (26.0 mN/m), indicating good thermal stability. In pH tests (2–12), the biosurfactant showed variations in activity, suggesting that its stability may be affected by pH changes. Regarding salinity, the compound demonstrated excellent stability, maintaining surface tension between 27.4 and 27.9 mN/m even at the highest NaCl concentrations tested (up to 12%).

Lima et al. [35] analyzed the surface tension stability of the biosurfactant from *C. lipolytica* cultivated in distilled water supplemented with molasses (4%), corn steep liquor (2.5%), and residual frying oil (2.5%) as substrates, and reported that pH variation did not result in significant changes in surface tension. Similarly, values remained stable at all evaluated temperatures, as well as at the different NaCl concentrations.

### 3.3. Characteristics of BS-AgNPs by Scanning Electron Microscopy (SEM)

According to standard E2456-06 of the American Society for Testing and Materials (ASTM), nanoparticles are defined as particles with dimensions between 1 and 100 nm. However some studies consider particles larger than 100 nm as nanoparticles (NPs), depending on the context and application [36]. Silver nanoparticles (AgNPs) are reduced forms of metallic silver with high potential for biological application, and can exhibit various shapes, such as spherical, flat, triangular, tetrahedral, prismatic, cubic, octahedral, and irregular, with variable size [37].

**Figure 2:**
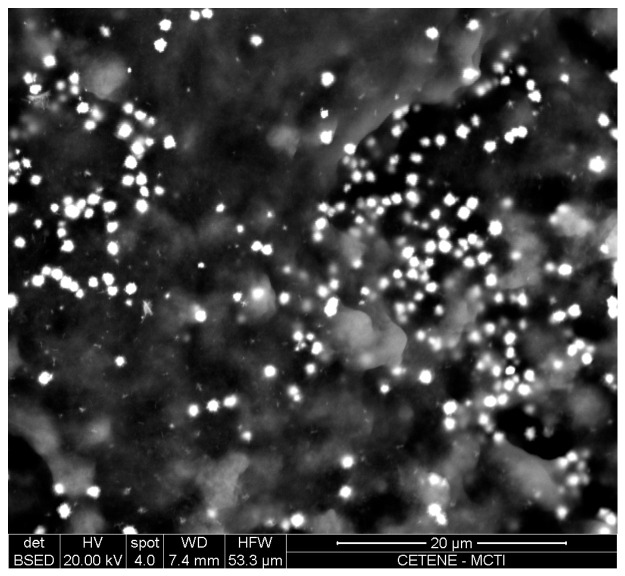
SEM image of silver nanoparticles stabilized with biosurfactant from *Candida glabrata* UCP 1002.

The morphological analysis of the silver nanoparticles stabilized with the biosurfactant produced by *Candida glabrata* UCP 1002 was performed by Scanning Electron Microscopy (SEM). In the image obtained (Figure 2), the formation of nanoparticles with predominantly spherical morphologies can be observed, presenting good distribution and a low degree of agglomeration, which suggests an effective stabilizing effect of the biosurfactant in the colloidal suspension.

The smallest particles of the produced BS-AgNPs presented sizes ranging from 100 to 240 nm, indicating a nanometric range consistent with the proposed biotechnological application. It is suggested the presence of nanoparticles in this range is compatible with antimicrobial activity, since smaller particles have a greater specific surface area, favoring the release of Ag^+^ ions and interaction with microorganisms. According to Coriolano et al. [37], the size of AgNPs directly influences nanoparticle activity, as smaller sizes increase the surface contact area of AgNPs with microorganisms.

The physical stability observed can be attributed to the action of the biosurfactant as a stabilizing agent, possibly through steric and/or electrostatic stabilization mechanisms, preventing agglomeration and promoting the maintenance of spherical morphology. According a study conducted by Lima et al. [35], SEM analysis revealed the presence of AgNPs as regions of high luminosity, an expected behavior given the high atomic number of silver relative to the organic matrix of the biosurfactant, which results in greater electron backscattering and, consequently, in characteristic compositional contrast. Similarly, they observed that AgNPs stabilized with biosurfactant from *C. lipolytica* UCP 0899 exhibited spherical morphology and good stability, possibly due to the action of the biosurfactant as a stabilizing agent, inhibiting aggregate formation.

The physical stability observed is compatible with the typical stabilization mechanisms of biosurfactants, which involve electrostatic interactions, hydrogen bonds, and hydrophobic interactions between biosurfactant molecules and the nanoparticle surface. Together, these mechanisms promote the formation of a stable coating around the AgNPs, inhibiting their agglomeration and preserving the spherical morphology over time [13].

### 3.4. Characteristics of BS-AgNPs by Energy Dispersive X-Ray Spectroscopy (ESD)

**Figure 3:**
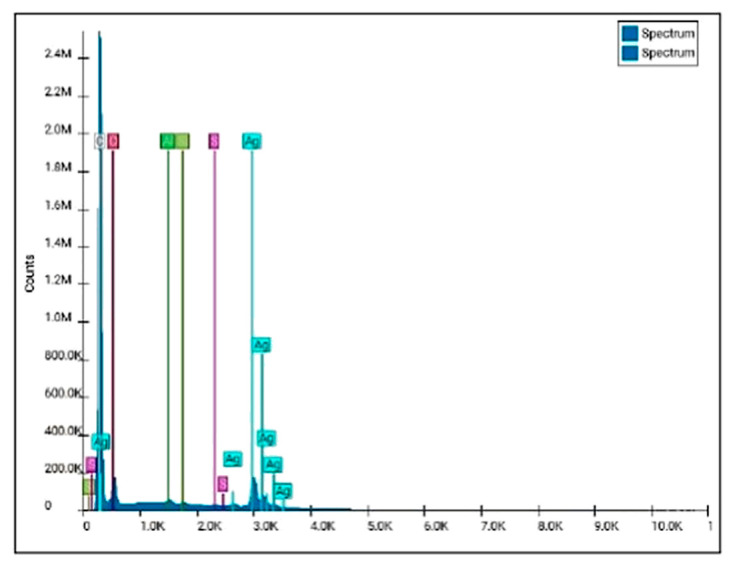
Elemental composition of the BS-AgNPS confirmed by EDS analysis.

Energy Dispersive X-ray Spectroscopy (EDS) analysis of the biosurfactant-stabilized silver nanoparticles confirmed the predominant presence of silver (Ag), evidenced by intense peaks observed mainly in the region near 3 keV, a typical characteristic of metallic AgNPs. In addition to silver, signals corresponding to carbon (C), oxygen (O), and sulfur (S) were also identified, suggesting the presence of organic compounds from the biosurfactant adsorbed onto the nanoparticle surface (Figure 3).

The carbon and oxygen peaks are likely associated with the organic functional groups present in the biosurfactant biomolecules, such as hydroxyls, carbonyls, and lipid chains, which act both in the reduction in Ag^+^ ions and in the stabilization of the nanoparticles. The presence of these elements reinforces the hypothesis of surface coating of the AgNPs by amphiphilic molecules produced by *Candida glabrata* UCP 1002, contributing to colloidal stability.

The sulfur (S) signal may be related to the presence of sulfur-containing groups derived from proteins, sulfur-containing amino acids, or metabolites associated with the biosurfactant. Recent studies have demonstrated that sulfur-containing compounds have high affinity for the silver surface and may participate in the chemical stabilization of nanoparticles through Ag–S interaction [38].

Similar results were reported by Salazar-Bryam et al. [39], who synthesized silver nanoparticles stabilized by rhamnolipids and observed, by EDS, a predominance of the silver element associated with the presence of oxygen and carbon from the biosurfactant adsorbed onto the particle surface. The authors highlighted that these organic compounds directly contribute to the stability and dispersion of AgNPs.

According to Markandeywar and Narang [40], that used biosurfactants as stabilizing agents in the green synthesis of AgNPs, the simultaneous presence of Ag, O, and C was identified in EDS spectra, associating these elements with the formation of a protective organic layer around the nanoparticles. This layer reduces aggregation processes and favors biomedical and antimicrobial applications.

In this regard, the high intensity of the silver signals observed in the spectrum confirms the efficiency of silver nitrate reduction and the successful formation of AgNPs. Furthermore, the absence of relevant metallic contaminants suggests high purity of the synthesized material.

### 3.5. Characteristics of BS-AgNPs by Zeta Potential and Particle Size by Dynamic Light Scattering (DLS)

**Figure 4:**
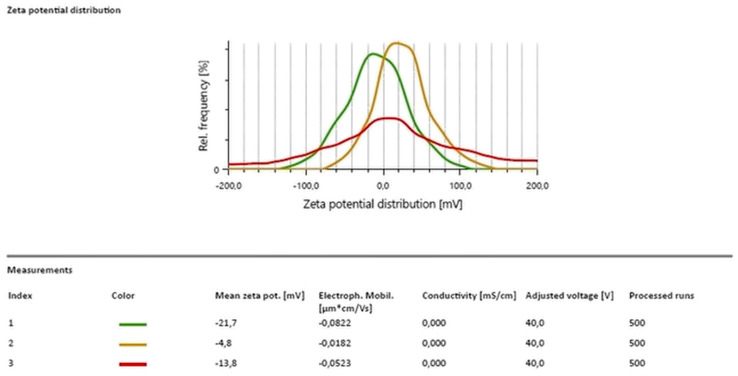
Zeta potential distribution of biosurfactant-stabilized AgNPs.

Zeta potential analysis of the biosurfactant-stabilized silver nanoparticles showed mean values of −21.7 mV, −4.8 mV, and −13.8 mV for the three evaluated samples, respectively (Figure 4). A predominance of negative surface charges was observed, indicating that the biosurfactant produced by *Candida glabrata* UCP 1002 acted as a stabilizing agent for the AgNPs, promoting electrostatic repulsion between the particles and reducing the tendency toward aggregation.

The zeta potential distribution graphs for the triplicates of the same sample showed similar profiles, but with small variations in the distribution of surface charges. The mean zeta potential values obtained were −21.7 mV, −13.8 mV, and −4.8 mV, indicating a predominance of negative charges on the nanoparticles stabilized by the biosurfactant. The replicate with a mean value of −21.7 mV showed a distribution more shifted toward negative values, suggesting greater electrostatic repulsion intensity and better colloidal stability. The assays with values of −13.8 mV and −4.8 mV, in turn, exhibited broader distributions closer to neutrality, which may indicate partial reduction in stability and a greater tendency toward nanoparticle aggregation.

In nanoparticulate systems, zeta potential values greater than ±30 mV are generally associated with high colloidal stability due to strong inter-particle repulsion. However, formulations stabilized by biosurfactants may present satisfactory stability even at intermediate zeta potential ranges, owing to the simultaneous contribution of steric and electrostatic mechanisms promoted by the surfactant biomolecules [41].

The results obtained in this study demonstrate the stability of the synthesized BS-AgNPs, which may have favored the maintenance of colloidal dispersion and contributed to the observed antimicrobial activity. These findings corroborate recent studies in the literature involving silver nanoparticles stabilized by biosurfactants. Lima et al. [35] observed a zeta potential of approximately −60 mV in AgNPs stabilized with biosurfactant from *Candida lipolytica*, indicating high colloidal stability and strong electrostatic repulsion between the particles.

Similar results were described by studies using rhamnolipids as stabilizing agents for AgNPs. Under different pH conditions, the authors observed zeta potentials ranging from −29.86 mV to −40.33 mV, demonstrating that biosurfactants can significantly increase nanoparticle stability and directly influence their size and colloidal behavior [39].

### 3.6. Antimicrobial Activity

The antimicrobial activity of the silver nanoparticles stabilized with the biosurfactant (BS-AgNPs) from *C. glabrata* UCP 1002 was evaluated against bacterial strains of *Enterobacter* sp., *Staphylococcus aureus*, *Escherichia coli*, and *P. aeruginosa* isolated from clinical samples from a hospital, and against the yeast *Candida albicans*. According to the data shown in Table 2, the evaluated biosurfactant demonstrated antimicrobial activity against different microorganisms, presenting variation in mean inhibition halos and inhibition percentages, which evidences its potential as a broad-spectrum antimicrobial agent.

Against *Enterobacter* sp., a Gram-negative bacterium of the family Enterobacteriaceae, a mean inhibition halo of 1.37 ± 0.05 cm and an inhibition percentage of 14.42% were observed. Despite the natural resistance of this group, the result indicates moderate activity of the biosurfactant, suggesting that it may be a complementary alternative to traditional antimicrobials. For *Escherichia coli*, also Gram-negative, the mean halo was 1.30 ± 0.1 cm and inhibition was 13.68%, indicating sensitivity similar to that of *Enterobacter* sp.

Regarding *Staphylococcus aureus*, a Gram-positive bacterium, the BS-AgNPs presented a mean halo of 1.67 ± 0.2 cm and an inhibition percentage of 17.58%, demonstrating greater sensitivity, possibly due to the peptidoglycan-rich cell wall structure, which is more vulnerable to the action of amphiphilic compounds.

*Pseudomonas aeruginosa*, recognized for its high resistance to multiple antibiotics and for forming biofilms, showed a significant response to BS-AgNPs, with a mean halo of 2.53 ± 0.3 cm and an inhibition percentage of 26.63%. This result is especially relevant, as it indicates that BS-AgNPs may interfere with the integrity of the outer membrane or the structure of biofilms.

**Figure 5:**
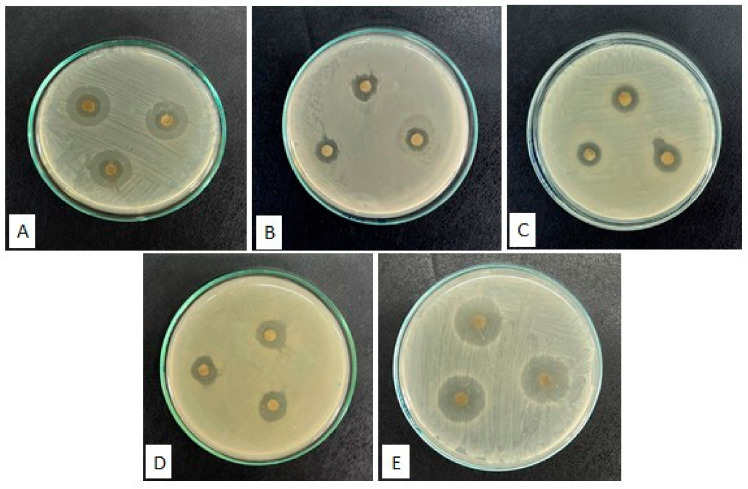
Formation of inhibition halos by silver nanoparticles stabilized with the biosurfactant (BS-AgNPs) from *C. glabrata* UCP 1002 against tested microorganisms: (**A**) *Pseudomonas aeruginosa*; (**B**) *Escherichia coli*; (**C**) *Enterobacter* sp.; (**D**) *Staphylococcus aureus*; (**E**) *Candida albicans*.

The better performance was observed against the yeast *Candida albicans*, with a mean halo of 2.67 ± 0.4 cm and an inhibition percentage of 28.11% (Figure 5). This expressive antifungal effect may be related to the ability of BS-AgNPs to interact with components of the fungal membrane, such as ergosterol, promoting destabilization and cell lysis. Therefore, the data suggest that BS-AgNPs has promising potential as an antimicrobial agent, particularly against microorganisms of clinical interest, such as *P. aeruginosa* and *C. albicans*. Figure 4 presents evidence of the antimicrobial activity of the biosurfactant through inhibition halos. According Castillo et al. [42], ergosterol, is an essential structural component of the fungal cell membrane, constitutes a primary target of antifungal compounds; its biosynthetic inhibition promotes the accumulation of sterol precursors, compromising membrane integrity and culminating in the loss of cell viability. In this sense, BS-AgNPs promote extensive disruption in *Candida albicans*, affecting surface morphology, cellular ultrastructure, membrane microenvironment, fluidity, ergosterol content, and fatty acid composition, thereby compromising multiple cellular targets associated with drug resistance and pathogenicity [43].

To establish a reliable parameter for comparing the antimicrobial activity of the biosurfactant-stabilized silver nanoparticles, parallel tests were performed using commercial antibiotics. The use of antibiotics as a positive control ensures the methodological validity of the microbiological assays.

The results obtained in the antimicrobial susceptibility assay revealed a varied sensitivity profile among the tested microorganisms against the evaluated commercial antibiotics. The most effective compounds were streptomycin (S300) and moxifloxacin (MXF 5), which produced the largest inhibition halos in the three Gram-negative bacterial strains. Streptomycin showed inhibition of 26.32% in *Enterobacter* sp., 25.26% in *S. aureus*, and 20.00% in *E. coli*, while moxifloxacin showed very similar results against *Enterobacter* (24.74%) and *S. aureus* (26.84%), although with lower efficacy against *E. coli* (10.53%) and *P. aeruginosa* (13.68%). Table 3 presents the results of the antimicrobial activity of commercial antibiotics against the tested microorganisms, expressed as mean halos and inhibition percentages.

Biosurfactants possess the ability to inhibit the growth of various pathogenic and environmental bacterial strains, as well as fungi, yeasts, algae, and viruses. Although the mechanism of action has not yet been fully elucidated, their antimicrobial activity is broadly attributed to the amphiphilic nature of these molecules, which allows for destabilization of the cell membrane. This interaction results in increased permeability, pore formation, leakage of metabolites, and eventual cell lysis. When associated with nanostructures, biosurfactants demonstrate enhanced activity, generally attributed to the greater binding affinity with the cell surface conferred by the surfactant properties. Furthermore, the specific action of these compounds against different microorganisms contributes to a synergistic effect, intensifying the antimicrobial efficacy of nanoformulations [44].

Durval et al. [45] synthesized AgNPs using the biosurfactant produced by *Bacillus cereus* UCP 1615 as a stabilizing agent in the synthesis. The antimicrobial potential of the BS-AgNPs was evaluated at different concentrations against three pathogenic fungi: *Aspergillus niger*, *Penicillium fellutanum*, and *Cladosporium cladosporioides*. The concentration of 16.50 µg/mL demonstrated high efficacy, promoting total inhibition (100%) of *P. fellutanum* growth and an 85% reduction in *A. niger* growth on potato dextrose agar medium.

Ferreira et al. [46] investigated the use of plant-derived surfactants, extracted from tangerine peels, as stabilizing agents in nanoparticle synthesis, aiming for an ecologically sustainable alternative to synthetic surfactants. The nanoparticles obtained demonstrated antimicrobial activity against *Escherichia coli* and *Staphylococcus aureus*.

Lima et al. [35] synthesized, in a simple and eco-friendly manner, silver nanoparticles (AgNPs) using the biosurfactant produced by *Candida lipolytica* UCP 0899 as a stabilizing agent. The stabilized nanoparticles were evaluated for their antimicrobial activity against bacterial isolates of *Pseudomonas aeruginosa*, *Staphylococcus aureus*, *Escherichia coli*, and *Enterobacter* sp., as well as fungal isolates of *Candida albicans* and *Aspergillus niger*. At a concentration of 16.50 µg/mL, the AgNPs demonstrated the ability to inhibit the growth of all tested microorganisms: *E. coli* (95%), *S. aureus* and *C. albicans* (90%), *A. niger* (85%), *Enterobacter* sp. (75%), and *P. aeruginosa* (71%).

Therefore, the literature data analyzed reinforce the findings of the present study, demonstrating that BS-AgNPs exhibit significant antimicrobial activity against a wide variety of microorganisms. The similarity in inhibition profiles confirms the potential of these sustainable approaches as promising alternatives in combating pathogens, especially in a scenario of growing resistance to conventional antimicrobials.

### 3.7. Cytotoxic Evaluation of the Biosurfactant

Cytotoxicity refers to the ability of a compound to inhibit the synthesis of macromolecules essential to the cell, resulting in structural and functional alterations that may lead to cell death. Cytotoxicity assays are in vitro methods used to evaluate the degree of cellular damage caused by a substance. These assays allow the identification of both cytostatic effects, which inhibit cell multiplication, and cytocidal effects, which promote cell death [47].

The results obtained in the cytotoxicity assay by the MTT method revealed important differences in the activity profile between the tested biosurfactant and the reference drug paclitaxel (Table 4). The biosurfactant produced by *C. glabrata* UCP 1002 presented a singular cytotoxic activity profile. Its action was considerably reduced against tumor cells, with inhibition of 10.77% for MCF-7 (human breast adenocarcinoma), 4.68% for HT-29 (human colon adenocarcinoma), and no activity against HL-60 (human promyelocytic leukemia). In contrast, it demonstrated low toxicity against normal Vero CCL-81 cells (African green monkey kidney epithelial cells), with 28.42% inhibition, which suggests greater biocompatibility.

Paclitaxel exhibited high cytotoxicity against the human tumor cell lines MCF-7, HT-29, and HL-60, with inhibition percentages of 94.75%, 89.77%, and 75.48%, respectively. However, it also demonstrated significant toxicity against the non-tumor Vero CCL-81 cells (75.33%), which evidences its low selectivity and potential to cause side effects in healthy tissues.

Liebmann et al. [48], through in vitro clonogenic assays, demonstrated that paclitaxel exhibits high cytotoxicity in eight human tumor cell lines, including MCF-7 (breast) and HT-29 (colon), with IC_50_ values ranging between 2.5 and 7.5 nM after 24 h of exposure; this study has become a classic reference for evaluating paclitaxel cytotoxicity in these cell lines. Paclitaxel demonstrates significant efficacy in the treatment of rapidly growing tumors, but faces a relevant limitation, its low tumor selectivity, which constitutes one of its main disadvantages in the clinical context [49].

Santos et al. [33] performed cytotoxic evaluation using the MTT assay on murine fibroblasts (L929) and demonstrated that the biosurfactant from *Candida sphaerica* did not exert relevant toxic effects on the tested cell line, with cell viability indices maintained above 90% across the entire range of evaluated concentrations. This behavior reflects a favorable biocompatibility profile, an attribute of great relevance for biomolecules intended for biotechnological purposes, since the absence of cytotoxicity against mammalian cells constitutes a fundamental requirement for their safe application in biomedical and industrial contexts.

Although the biosurfactant did not demonstrate direct cytotoxicity against the tested tumor cell lines, its low toxicity in normal cells represents a desirable characteristic in strategies aimed at reducing the adverse effects typical of conventional chemotherapeutic agents [50]. In this context, its use may be promising as a component of controlled release systems or as an adjuvant agent in therapeutic formulations [51,52], reinforcing its potential for safe biomedical applications.

## 4. Conclusions

The biosurfactant produced by *Candida glabrata* UCP 1002 demonstrated high efficiency in reducing surface tension, as well as physicochemical stability under different conditions of temperature, pH, and salinity, evidencing robustness for biotechnological applications. The use of agroindustrial residues as substrates reinforces the sustainable and economically viable nature of the production process.

The synthesis of biosurfactant-stabilized silver nanoparticles occurred efficiently, resulting in predominantly spherical AgNPs with low agglomeration and elemental composition compatible with the presence of metallic silver coated by organic biomolecules from the biosurfactant. Zeta potential analyses indicated a predominance of negative surface charges and colloidal stability, confirming the role of the biosurfactant as a stabilizing agent.

The BS-AgNPs exhibited antimicrobial activity against Gram-positive bacteria, Gram-negative bacteria, and the yeast *Candida albicans*, with particular emphasis on the results obtained against *Pseudomonas aeruginosa* and *C. albicans*, microorganisms of high clinical relevance. The results suggest the potential application of the nanoformulations in the development of antimicrobial alternatives in the face of increasing microbial resistance.

Future investigations should explore the potential of BS-AgNPs against biofilm-forming and multidrug-resistant clinical strains, which represent a growing global health threat. The evaluation of synergistic effects between BS-AgNPs and conventional antibiotics may also contribute to the development of more effective therapeutic strategies. Given the broad biomedical potential of silver nanoparticles, future studies could investigate the application of BS-AgNPs in wound healing formulations, antimicrobial coatings for medical devices, and drug delivery systems. Additionally, in vivo studies are encouraged to validate the efficacy and biosafety of these nanoformulations, supporting their translational potential toward clinical and industrial applications.

## Figures and Tables

**Table 1:** Stability of the biosurfactant produced by *Candida glabrata* UCP 1002.

**Temperature (°C)**	**Surface Tension mN/m**	**pH**	**Surface Tension** **mN/m**	**NaCl (%)**	**Surface Tension mN/m**
		2	34.9 ± 0.04	2	33.9 ± 0.8
0	34.1 ± 0.04	4	34.4 ± 0.04	4	32.8 ± 0.2
5	34.2 ± 0.03	6	29.4 ± 0.07	6	32.9 ± 0.6
70	32.6 ± 0.06	8	31.4 ± 0.01	8	33.8 ± 0.1
100	32.2 ± 0.08	10	32.4 ± 0.1	10	35.2 ± 0.2
		12	34.0 ± 0.02	12	34.7 ± 0.5

**Table 2:** Antimicrobial activity of the silver nanoparticles stabilized with the biosurfactant (BS-AgNPs) from *C. glabrata* UCP 1002 against different microorganisms.

Microorganisms	Medium Halo (cm)	% Inhibition
*Enterobacter* sp.	1.37 ± 0.05	14.42
*Staphylocaccus aureus*	1.67 ± 0.2	17.58
*Escherichia coli*	1.30 ± 0.1	13.68
*Pseudomona aeruginosa*	2.53 ± 0.3	26.63
*Candida albicans*	2.67 ± 0.4	28.11

**Table 3:** Evaluation of the antimicrobial activity of commercial antibiotics.

Microorganisms	Streptomycin 300 µg (S300)	Moxifloxacin 5 µg (MXF 5)	Cefotaxime 5 µg (CTX 5)	Nitrofurantoin 300 µg (F300)	Cefpodoxime 10 µg (CPD 10)
*Enterobacter* sp.					
**Medium Halo (cm)**	2.5 ± 0.141	2.3 ± 0.071	-	0.5 ± 0.707	-
**% Inhibition**	26.32	24.74	-	5.26	-
*Staphylocaccus aureus*					
**Medium Halo (cm)**	2.4 ± 0.141	2.5 ± 0.071	-	1.7 ± 0.141	-
**% Inhibition**	25.26	26.84	-	17.89	-
*Escherichia coli*					
**Medium Halo (cm)**	1.9 ± 0.424	1.0 ± 1.414	-	0.5 ± 0.778	-
**% Inhibition**	20.00	10.53	-	5.79	-
*Pseudomona aeruginosa*					
**Medium Halo (cm)**	1.3 ± 1.838	1.3 ± 1.838	0.5 ± 0.778	0.9 ± 1273	0.6 ± 0.849
**% Inhibition**	13.68	13.68	5.79	9.47	6.32

**Table 4:** Cytotoxic activity of the test substances on cancerous and non-cancerous cell lines.

Test Products	MCF-7	HT-29	HL-60	Vero CCL-81
Biosurfactant	10.77 ± 1.12	4.68 ± 0.33	0.00 ± 0.00	28.42 ± 2.64
Paclitaxel	94.75 ± 1.1	89.77 ± 1.9	75.48 ± 4.08	75.33 ± 4.02

## Data Availability

The original contributions presented in this study are included in the article. Further inquiries can be directed to the corresponding author.
